# The high level of IL-1β in the serum of ACLF patients induces increased IL-8 expression in hUC-MSCs and reduces the efficacy of hUC-MSCs in liver failure

**DOI:** 10.1186/s13287-023-03455-9

**Published:** 2023-08-30

**Authors:** Yong-Hong Wang, Meng-Lan Wang, Ya-Chao Tao, Dong-Bo Wu, En-Qiang Chen, Hong Tang

**Affiliations:** 1 Center of Infectious Diseases, West China Hospital of Sichuan University, Chengdu, 610041 Sichuan China; 2 Division of Infectious Diseases, State Key Laboratory of Biotherapy and Center of Infectious Diseases, West China Hospital, Sichuan University, Chengdu, China

**Keywords:** Liver failure, Mesenchymal stem cells, Serum, Interleukin-8, Interleukin-1β

## Abstract

**Background:**

Stem cells play a therapeutic role mainly through immunoregulation. However, the immunomodulatory function of stem cells may be affected by inflammation-related factors in patients’ serum. Therefore, this study aims to investigate the possible mechanism by which acute-on-chronic liver failure (ACLF) patient serum influences the efficacy of hUC-MSCs.

**Methods:**

The serum of surviving and dead ACLF patients was collected to culture hUC-MSCs in vitro, and the hUC-MSCs cultured in the serum of ACLF patients were used to treat acute liver failure (ALF) rats. The therapeutic effect on the rats was evaluated by a survival curve, the transaminase level and liver histopathology. The expression of cytokines in hUC-MSCs was detected by Q-PCR and ELISA.

**Results:**

Serum pretreatment reduced the therapeutic effect of hUC-MSCs on ALF, especially pretreatment in the serum from dead ACLF patients. After hUC-MSCs were cultured in the serum of surviving or dead ACLF patients, the most differentially expressed factor was IL-8. Interfering with the expression of IL-8 in hUC-MSCs can improve the therapeutic effect of hUC-MSCs on ALF. The high level of IL-1β in the serum of dead ACLF patients causes the increased expression of IL-8 in hUC-MSCs through the activation of the NF-κB signaling pathway. Meanwhile, we found that the neutralizing IL-1β in serum from dead ACLF patients can improve the therapeutic effect of hUC-MSCs on ALF.

**Conclusion:**

The high level of IL-1β in ACLF serum can promote the expression of IL-8 in hUC-MSCs through the NF-κB signaling pathway, thus reducing the effect of hUC-MSCs on ALF.

**Supplementary Information:**

The online version contains supplementary material available at 10.1186/s13287-023-03455-9.

## Introduction

Liver failure is a common clinical severe liver disease syndrome with rapid progression and high mortality. Acute-on-chronic liver failure (ACLF) is the most common type of liver failure in China [1]. At present, the main treatment methods for liver failure include internal medicine treatment, artificial liver and liver transplantation. Liver transplantation is the most effective treatment, but due to the lack of donor liver, high cost of transplantation and serious immune rejection after transplantation, its wide application is limited [2, 3]. Therefore, finding new effective treatments for liver failure remains an urgent clinical problem. In recent years, stem cells have been proven to be useful in the treatment of many diseases [4].

Stem cells are a kind of cells with self-replication, high proliferation and multidifferentiation potential that have the functions of anti-inflammation, anti-apoptosis, immunomodulation, secretion of growth factors, promotion of angiogenesis and tissue repair [5–7]. At present, a variety of stem cells have been reported to be used in the treatment of liver diseases, among which mesenchymal stem cells (MSCs) are the most widely used stem cells due to their advantages such as wide sourcing, easy access, low immunogenicity and no ethical restrictions [8]. Previous studies have shown that stem cells are expected to be an effective treatment for liver failure, but the clinical efficacy is unstable. At present, most studies focus on the mechanism of how MSCs can effectively treat liver failure, but the factors that may influence MSCs in treating liver failure remain unclear. MSCs play a therapeutic role in various diseases mainly through immunomodulatory functions, and this immunomodulatory function will be affected by various inflammation-related factors in patients’ serum. High concentrations of IFN-γ and TNF-α can induce the high expression of IDO in MSCs and then inhibit the proliferation of T cells. However, the high concentrations of IL-10 and TGF-β can inhibit the expression of IDO in MSCs and weaken the proliferation inhibition ability of MSCs on T cells [9–11]. Therefore, inflammation-related factors in different individuals can change the immunomodulatory function of MSCs, which may lead to differences in MSCs efficacy.

In liver failure, the production of a large number of inflammatory mediators promotes the activation of immune cells and the secretion of inflammatory cytokines, which aggravates the death of hepatocytes [12, 13]. The inflammatory factors that have been reported to be associated with poor prognosis of liver failure include TNF-α, IL-1β,and IL-6, etc. [14–16]. Inflammatory factors in patients’ serum are likely to affect the immunomodulatory function of MSCs, thus enhancing or weakening the efficacy of MSCs in the treatment of liver diseases [17]. Therefore, this study aims to investigate the possible mechanism by which ACLF patient serum influences the efficacy of MSCs, and to provide evidence for how to choose ACLF patients who are suitable for stem cell therapy.

## Materials and methods

### Collection of serum samples and patient data

From March 2018 to May 2019, serum samples from 27 healthy people, 52 chronic hepatitis B (CHB) patients and 145 ACLF patients were collected from West China Hospital of Sichuan University. All collected serum was stored at − 80 °C. At the same time, the patients’ sex, age, clinical diagnosis and the liver and kidney function, routine blood, coagulation routine, alpha fetoprotein and other examination results were recorded. Previous studies have shown that the death peaks of ACLF patients occur within 30 days after admission; thus, the judgment time of survival and death of ACLF patients in this study was within 30 days after admission.

### Culture and identification of hUC-MSCs

Human umbilical cord MSCs (hUC-MSCs) in our study were provided by Hui Rong Tong Chuang Biological Technology, Ltd. The complete culture medium of hUC-MSCs was Dulbecco’s modified Eagle’s medium (DMEM) containing 20% fetal bovine serum (Gibco, USA), 100 units/ml penicillin, and 100 μg/ml streptomycin(Gibco, USA). The cells were cultured in an incubator at 37 °C and 5% CO_2_, and the culture medium was changed every 3 ~ 4 days. Before the hUC-MSCs were cultured with serum from ACLF patients, the ACLF serum stored in a − 80 °C refrigerator was melted at room temperature and filtered with a 0.22 µm filter. The serum of the surviving or dead ACLF patients used in this experiment was a mixture of serum from 89 surviving or 56 dead ACLF patients, respectively. When hUC-MSCs were grown to 70–80% confluence in 75 cm^2^ culture flasks, they were cultured for 48 h after adding 10 ml of culture medium containing 50%(v/v) ACLF serum.

The identification of hUC-MSCs is based on morphology, cell marker expression and differentiation potential. We used flow cytometry (BD Accuri™ C6) to detect the expression of CD90, CD105, CD34 and CD45 in hUC-MSCs. All antibodies were PE labeled and purchased from BD company. The differentiation potential of hUC-MSCs was verified with adipogenic, osteogenic, and hepatic differentiation kits (Cyagen Biosciences Inc, China). Differentiation was induced according to the manufacturer’s protocol. Adipogenesis and osteogenesis were assessed by oil red O and alizarin red staining, respectively. The hepatogenic differentiation of hUC-MSCs was detected by glycogen staining, indocyanine green uptake and the expression of hepatocyte specific markers (AFP, ALB and CK-18).

### HUC-MSCs proliferation assay

The cell number was adjusted to 5 × 10^4^/ml, and 100 µl of cell suspension was added to each well of the 96-well plate. The 96-well plate was placed in a 37 ℃, 5% CO_2_ incubator overnight. After discarding the old culture medium, 100 µl of culture medium containing 50% (v/v) ACLF serum was added to each well for 24 h. Each well was treated with 20 µl MTT solution (5 mg/ml) for 4 h, and 150 µl DMSO was added after discarding the culture medium. Finally, the absorbance value was detected with a microplate reader.

### HUC-MSCs IL-8 interference and serum IL-1β neutralization

When hUC-MSCs were grown to 70–80% confluence in 75 cm^2^ culture flasks, 10 ml of fresh culture medium was replaced and IL-8 siRNA transfection complex was added. Ten milliliters of culture medium require 1.07 ml of transfection complex (1ml PepMute™ transfection buffer (SignaGen, USA) + 40 µl PepMute™ reagent (SignaGen, USA) + 30 µl IL-8 siRNA). IL-1β neutralizing antibody (R&D, USA) was added to the medium containing 50% (v/v) ACLF serum, and the final working concentration of IL-1β neutralizing antibody was 20 ng/ml.

### Animal models and cell transplantation

Two hundred and twenty-five male Sprague–Dawley rats (weight 180 ~ 220 g) were obtained from Cheng Du Dossy Experimental Animals Co, Ltd. (Chengdu, China). Rats were raised in an animal laboratory with stable temperature and humidity and allowed free access to chow and water. All rats were placed in a cage and randomly sorted into each group (15 rats in each group).The rat model of acute liver failure was established by a single intraperitoneal injection of 700 mg/kg d-galactosamine (Sigma, USA) and 20 µg/kg lipopolysaccharide (Sigma, USA). hUC-MSCs transplantation was performed 2 h after d-GalN/LPS treatment. The method of hUC-MSCs transplantation was performed by injecting 3 × 10^7^ cells/kg from the tail vein, and the cell suspension was slowly injected within 3 ~ 5 min. The order of treatment and measurement of the different groups is random. The outcome was evaluated by comparison of 48h survival rate, transaminase level and liver histopathology in ALF rats. Euthanasia was performed by intraperitoneal injection of 200 mg/kg sodium pentobarbital in a volume of 2 ml/kg.

### Biochemical analysis and HE staining

Blood samples were obtained from each rat and centrifuged for 15 min at 3000 rpm and serum was collected. Concentrations of alanine aminotransferase (ALT) and aspartate aminotransferase (AST) were measured with an automated biochemical analyzer (Abbott, USA). Each liver sample was fixed in 4% paraformaldehyde for 24 h before histological analysis. Fixed liver samples were cut into small pieces, dehydrated, paraffin-embedded, and cut into sections 5 μm thick. Sections were stained with hematoxylin and eosin for pathological assessment. All microscope images were collected by Olympus CKX53 microscope and ACDSee software in this study.

### Real-time PCR analysis

Total RNA was isolated from hUC-MSCs using TRIzol extraction (Invitrogen, USA). Quantitative real-time (qRT)-PCR was performed using a LightCycler FastStart DNA Master PLUS SYBR Green I kit (Roche, Switzerland). The primer sequences (Additional file 1: Table S1–S3) specific to target genes were synthesized (Tsingke, China). All samples were amplified in triplicate.

### Cytokine level measurement

Serum samples from healthy people, CHB patients and ACLF patients were collected, and interleukin-8 (IL-8), tumor necrosis factor α (TNF-α) and interleukin-1β (IL-1β) levels were analyzed using a human IL-8, TNF-α and IL-1β ELISA kit (NeoBioscience, China). Tests were performed according to the manufacturer’s protocol and every sample was run in triplicate. Concentration was determined by comparison with the standard curve.

### Western blot analysis

Western blot analysis was used to detect the P-NF-κB-P65, NF-κB-P65 and IL-8 activity of hUC-MSCs after IL-1β or ACLF serum (from dead patients) preconditioning. The cells were harvested, and total proteins were extracted in cell lysis buffer with protease and phosphatase inhibitors. Proteins were quantified using a protein assay (Bio-Rad, USA), and 50 μg of protein was separated by SDS-PAGE. Membranes were blocked in TBS, 0.1% Tween 20, and 5% BSA for 2 h before overnight incubation with monoclonal rabbit antibodies against human P-NF-κB-P65, NF-κB-P65 (CST, USA) and IL-8 (R&D, USA) diluted 1:1000. The membranes were incubated for 1 h in goat anti-rabbit IgG (eBioscience, USA) at a dilution of 1:2000.

### Statistical analysis

The data conforming to the normal distribution are expressed as the mean ± standard deviation, the data not conforming to the normal distribution are expressed as median (*p*25–*p*75), and the classified variables were expressed as numbers and percentages. A T test or analysis of variance was used for statistical analysis of the variables in accordance with normal distribution, and Mann–Whitney *U* test was used for the variables and classified variables in accordance with normal distribution. SPSS 17.0 software was used for statistical analysis. *P* < 0.05 (two tailed) was considered statistically significant.

## Results

### Basic information and clinical data of patients

In this study, serum was collected from 27 healthy people, 52 CHB patients and 145 ACLF patients, and the clinical data were collected for statistical analysis. After the normality test, the data conforming to the normal distribution were expressed as the mean ± standard deviation, and the data not conforming to the normal distribution were expressed as the median. The clinical data showed statistically significant differences in INR, TBIL, ALT, ALB, PLT and AFP among the three groups (*P* < 0.05) (Table 1). At the same time, the basic clinical data of ACLF patients who survived or died were further analyzed, and the differences in INR, TBIL and AFP between the two groups were statistically significant (*P* < 0.05) (Table 2).

**Table 1:** Basic clinical data of healthy people, CHB patients and ACLF patients

Groups	Normal people (*n* = 27)	CHB patients (*n* = 52)	ACLF patients (*n* = 145)	*P *value
Age (years)	35 ± 15	38 ± 11	48 ± 12	0.095
Sex, male /female	19/8	38/14	116/29	0.085
INR	0.97 ± 0.09	1.15 ± 0.16	2.19 ± 0.79	< 0.001
TBiL (μmol/L)	9.48(5.95–12.45)	145.89(42.4–222)	406.74(281.8–508.5)	< 0.001
ALT (IU/L)	21(16–36)	220(90–856)	99(51–262)	0.001
ALB(g/L)	46.73 ± 4.51	36.98 ± 4.8	31.96 ± 3.46	< 0.001
Cr (μmol/L)	74.14 ± 24.14	71.83 ± 12.43	87.88 ± 24.41	0.104
WBC(× 10^9^/L)	6.99 ± 2.45	5.44 ± 1.69	7.12 ± 3.84	0.093
PLT(× 10^9^/L)	195.6(119.5–245)	176.52(126–232)	95.23(58–126)	< 0.001
AFP(ng/ml)	2.95(2.5–6)	39.42(4.74–116.69)	54.67(13.25–254.78)	< 0.001

**Table 2:** Basic clinical data of survival and death patients with ACLF

Groups	Survival (*n* = 89)	Death (*n* = 56)	*P* value
Age (years)	47 ± 11	53 ± 13	0.232
Sex, male /female	72/17	47/9	0.112
INR	2.17 ± 0.8	2.67 ± 0.92	< 0.001
TBiL (μmol/L)	411 (304.8–505.1)	477.31 (371.4–562.5)	< 0.001
ALT (IU/L)	92(57–231)	113(63–284)	0.176
ALB(g/L)	31.69 ± 4.03	32.47 ± 3.06	0.342
Cr (μmol/L)	86.53 ± 22.68	97.2 ± 24.4	0.157
WBC(× 10^9^/L)	7.05 ± 2.7	7.68 ± 2.12	0.253
PLT(× 10^9^/L)	95 (60–133.5)	85.7 (43.5–116.5)	0.084
AFP(ng/ml)	130.78 (7.6–140.5)	105.71 (6.8–128.1)	0.023

### Identification of hUC-MSCs

Under normal culture conditions, hUC-MSCs grew adherently in plastic bottles, with clear cell morphology and epithelioid or spindle arrangement (Additional file 1: Fig. S1A). After differentiation was induced using a commercial stem cell osteoblast, adipoblast, and hepatoblast differentiation kit, hUC-MSCs were induced to differentiate into osteoblasts, adipocytes (Additional file 1: Fig. S1B), and hepatocyte like cells (Additional file 1: Fig. S1C, D). The percentage of hUC-MSCs expressing the stem cell surface markers CD90 and CD105 was 99.9% and 97.6%, respectively, and the percentage of negative cell surface markers CD34 and CD45 was 1.1% and 1%, respectively (Additional file 1: Fig. S1E).

### The serum of dead ACLF patients reduces the therapeutic effect of hUC-MSCs on ALF

**Fig. 1:**
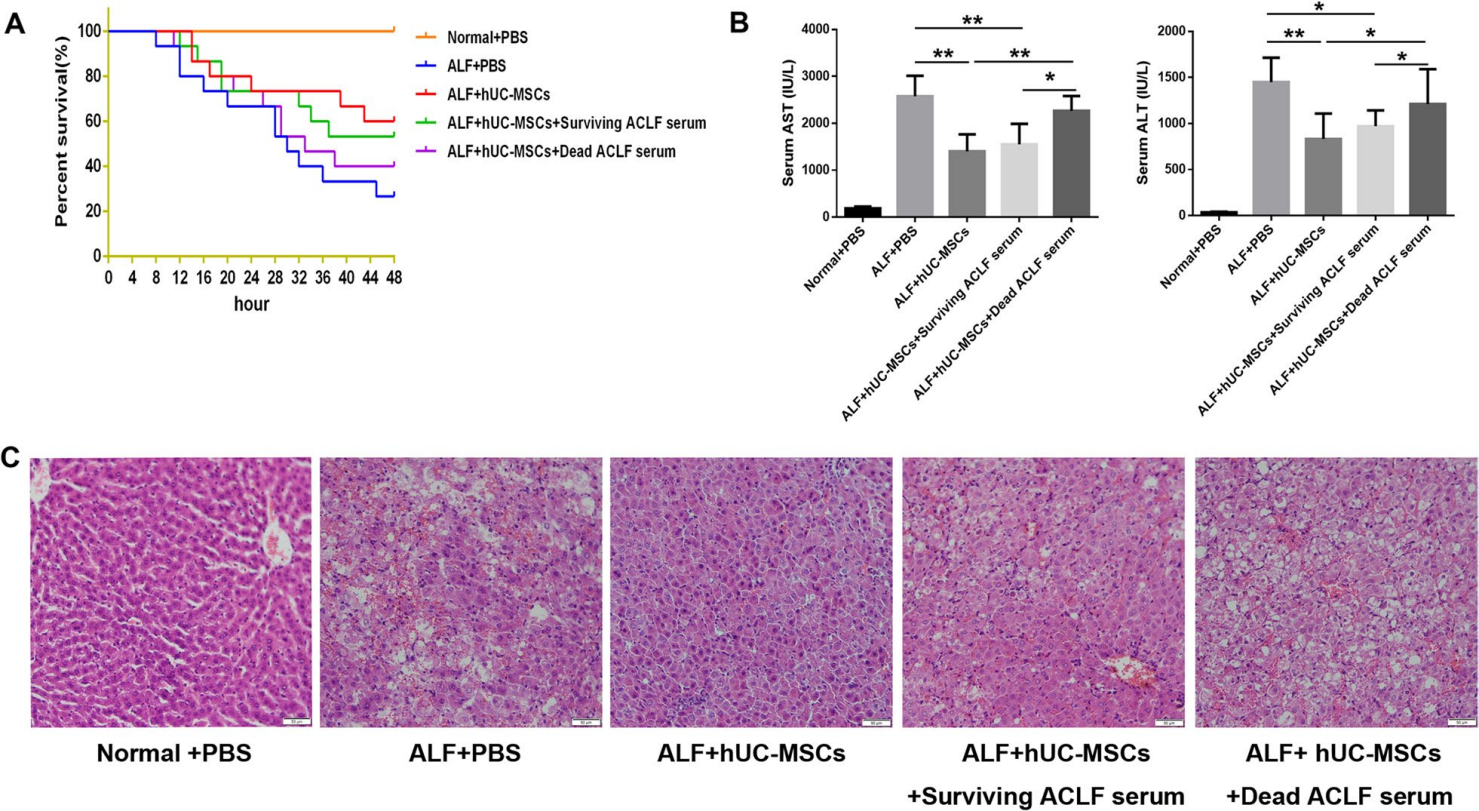
Effect of hUC-MSCs cultured in serum from surviving or dead ACLF patients on the treatment of ALF in rats. **A **Survival curves of different hUC-MSCs treatment groups (*n* = 15). **B** Serum ALT and AST levels of rats in different treatment groups (*n* = 4 ~ 9). **C** HE staining was used to observe the pathological changes in liver tissue. Scale bar = 50 μm. *P < 0.05; **P < 0.01 (DPI:300)

In the rat ALF model established by d-GalN/LPS 48 h after injection of PBS, hUC-MSCs, hUC-MSCs cultured in the serum of surviving ACLF patients and hUC-MSCs cultured in the serum of dead ACLF patients, the survival rate (surviving/total) of ALF rats in each group was 4/15 (26.7%), 9/15 (60%), 8/15 (53%) and 5/15 (33.3%), respectively (Fig. 1A). Liver function ALT/AST (IU/L) was 1449 ± 108.1/2574 ± 179.4, 833.0 ± 103.7/1402 ± 148.5, 971.1 ± 94.68 /1553 ± 138.3 and 1211 ± 143.3/2267 ± 129.2, respectively (Fig. 1B). The hUC-MSCs cultured under normal conditions and in the serum of surviving ACLF patients significantly reduced hepatocyte necrosis, sinus congestion and inflammatory cell infiltration in ALF liver tissue, but the hUC-MSCs cultured in the serum of dead ACLF patients did not alleviate the liver tissue damage (Fig. 1C). These results suggest that hUC-MSCs cultured under normal conditions can significantly reduce liver injury in ALF rats. The hUC-MSCs cultured in the serum of dead ACLF patients significantly aggravated the liver injury in ALF rats. However, the hUC-MSCs cultured in the serum of surviving ACLF patients have therapeutic effects similar to those of hUC-MSCs cultured under normal conditions.

### The serum of dead ACLF patients significantly increased the expression of IL-8 in hUC-MSCs

**Fig. 2:**
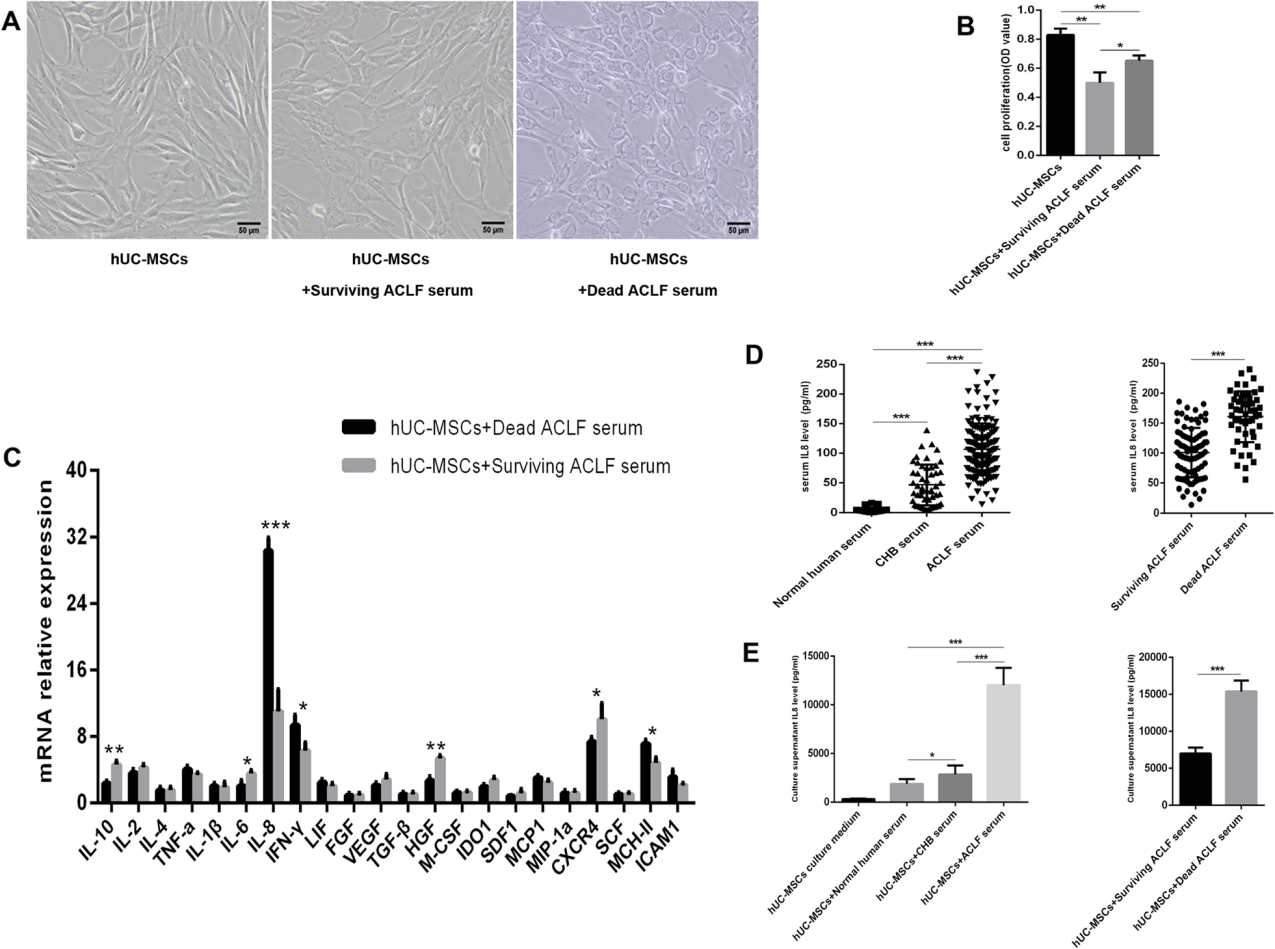
Serum from ACLF patients can increase the expression of IL-8 in hUC-MSCs. **A** Morphology of hUC-MSCs cultured in the serum of surviving or dead ACLF patients. **B** Proliferation of hUC-MSCs cultured in the serum of surviving or dead ACLF patients (*n* = 3). **C** Changes in the mRNA expression of 22 factors in hUC-MSCs cultured in the serum of surviving or dead ACLF patients. **D** ELISA was used to detect the expression of IL-8 in serum. **E** ELISA was used to detect the expression of IL-8 in the supernatant of hUC-MSCs. Scale bar = 50 μm. *P < 0.05; **P < 0.01; ***P < 0.001 (DPI:300)

After ACLF serum culture, the cytoplasmic granule like substances of hUC-MSCs increased, and the ACLF serum inhibited the proliferation of stem cells, especially the serum of surviving patients (Fig. 2A, B). The composition of ACLF serum is complex, and the expression level of various factors of hUC-MSCs may change under the influence of serum from different ACLF patients. Therefore, Q-PCR was used to detect the expression levels of 22 factors in hUC-MSCs cultured in the serum of surviving and dead ACLF patients. The mRNA expression levels of 22 factors in hUC-MSCs cultured in the serum of dead ACLF patients were compared with those in hUC-MSCs cultured in the serum of surviving ACLF patients. The mRNA expression levels of IL-10, IL-6, IL-8, IFN-γ, HGF, CXCR4 and MCH-II showed statistically significant differences between the two groups (*P* < 0.05), while the expression of IL-8 showed the most significant difference between the two groups(Fig. 2C).The expression level of IL-8 was gradually increased in the serum of healthy people, CHB patients and ACLF patients, and the expression of IL-8 was statistically significant among all groups (*P* < 0.05) (Fig. 2D), suggesting that the level of IL-8 in serum was gradually increased with the aggravation of the disease. At the same time, after hUC-MSCs were cultured with the serum of healthy people, CHB patients and ACLF patients, the detection showed that the level of IL-8 in the supernatant was gradually increased, and the expression of IL-8 was statistically significant among all groups (*P* < 0.05) (Fig. 2E), suggesting that the expression of IL-8 increased with the aggravation of the disease after hUC-MSCs was cultured in serum.

### Interference with the expression of IL-8 in hUC-MSCs improved the therapeutic effect of hUC-MSCs on ALF

**Fig. 3:**
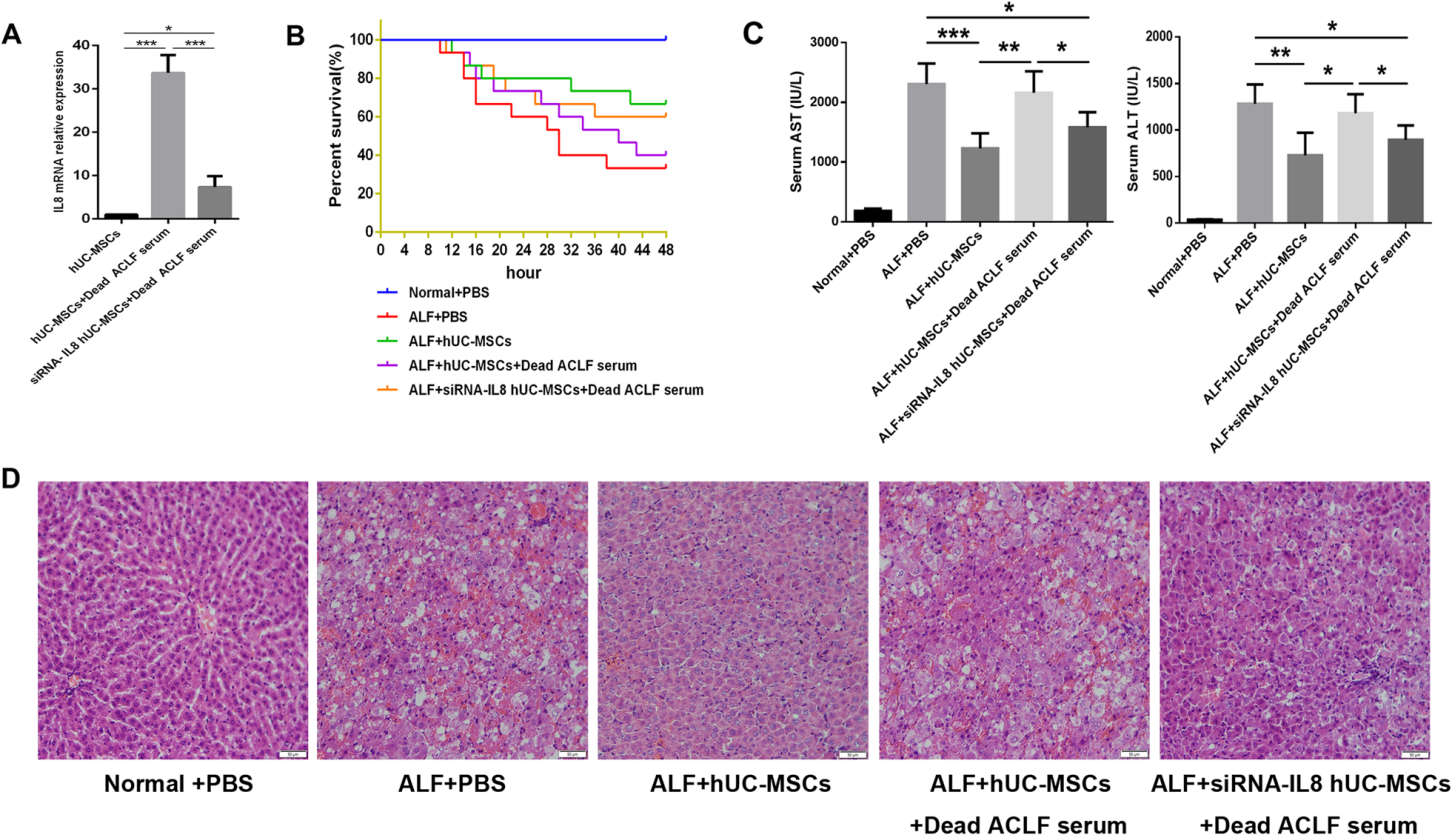
Effect of IL-8 on hUC-MSCs therapy for ALF. **A**The inhibitory effect of siRNA on IL-8 expression in hUC-MSCs. **B** Survival curves of different hUC-MSCs treatment groups (*n* = 15). **C** Serum ALT and AST levels of rats in different treatment groups (*n* = 5 ~ 10). **D** HE staining was used to observe the pathological changes in liver tissue. Scale bar = 50 μm.*P < 0.05; **P < 0.01; ***P < 0.001(DPI:300)

To investigate the effect of IL-8 on hUC-MSCs in the treatment of liver failure, a rat ALF model was established by d-GalN/LPS to further investigate the effect of IL-8 on liver failure. IL-8 expression of hUC-MSCs cultured in the serum of dead ACLF patients was significantly reduced by siRNA(Fig. 3A). In the rat ALF model, after the injection of PBS, hUC-MSCs, hUC-MSCs cultured in the serum of dead ACLF patients and siRNA-IL-8 hUC-MSCs cultured in the serum of dead ACLF patients, the survival rate (surviving/total) of ALF rats was 5/15(33.3%), 10/15(66.7%), 6/15(40%) and 9/15(60%), respectively (Fig. 3B). Liver function ALT/AST (IU/L) was 1282 ± 78.41/2307 ± 129.8, 728.9 ± 85.34/1230 ± 88.61, 1075 ± 67.52/2161 ± 127.9, and 824.4 ± 51.71/1584 ± 84.11, respectively (Fig. 3C). The pathological changes of liver tissue in each group are shown in the figure (Fig. 3D). The elevated expression of IL-8 in hUC-MSCs may weaken the therapeutic effect on ALF in rats.

### IL-1β in the serum of ACLF patients increased the level of IL-8 in hUC-MSCs

**Fig. 4:**
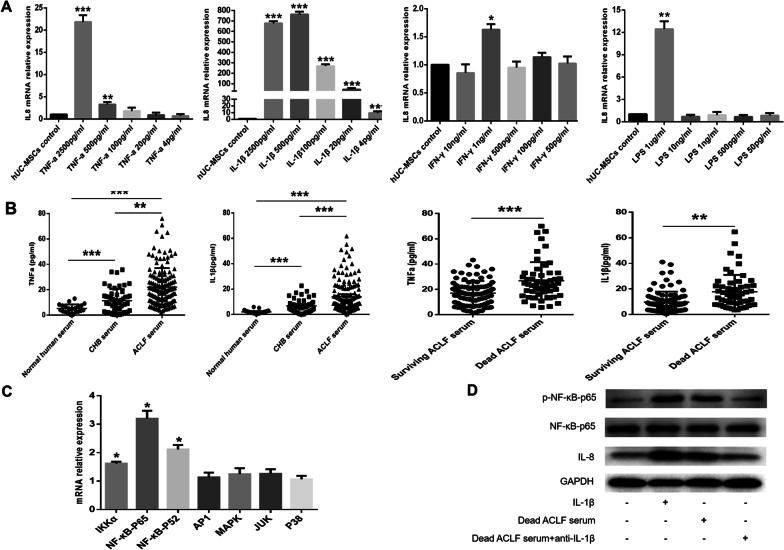
IL-1β in the serum of ACLF patients can increase the level of IL-8 in hUC-MSCs. **A** Effects of TNF -α, IL-1β, LPS and IFN-γ on the expression of IL-8 in hUC-MSCs. **B** The expression levels of IL-1β and TNF-α in the serum of different groups were detected by ELISA. **C** After IL-1β stimulation of hUC-MSCs for 24 h, changes in the pathways related to IL-8 expression in hUC-MSCs were detected by Q-PCR. **D** The effect of IL-1β on the NF-κB signaling pathway and IL-8 expression in hUC-MSCs was detected by Western blotting. Full-length blots are presented in supplementary figure s2. *P < 0.05; **P < 0.01; ***P < 0.001 (DPI:300)

To investigate the reasons for the increased expression of IL-8 in hUC-MSCs induced by ACLF serum, the KEGG pathway was used to identify the factors (including TNF-α, IL-1β, LPS and IFN-γ) that can induce the expression of IL-8. The expression of IL-8 in hUC-MSCs after 24 h of stimulation by the four factors was detected by Q-PCR to identify the factors that can stimulate the expression of IL-8 in hUC-MSCs. TNF-α can induce the expression of IL-8 in hUC-MSCs, but the induction effect is weak. The concentration of TNF-α at 500 pg/mL increased the relative expression level of IL-8 mRNA by 3.3-fold (Fig. 4A). IL-1β had a strong induction effect on the expression of IL-8 in hUC-MSCs, and IL-1β 4 pg/ml increased the relative expression level of IL-8 mRNA by 4.2-fold (Fig. 4A). However, high concentrations of LPS and IFN-γ induced a slight increase in the expression of IL-8 in hUC-MSCs (Fig. 4A). Because TNF-α and IL-1β had a strong induction effect on the expression of IL-8 in hUC-MSCs, the expression levels of TNF-α and IL-1β in the serum of each group were further detected. The serum levels of TNF-α and IL-1β were gradually increased in healthy people, CHB patients and ACLF patients, and the median concentrations of TNF-α and IL-1β in the serum of ACLF patients were 22.1 (10.5–59.6) pg/mL and 13.9 (6.5–41.4) pg/mL, respectively (Fig. 4B).

Further analysis of the serum levels of TNF-α and IL-1β in surviving and dead ACLF patients showed that the low concentration of TNF-α in the serum of ACLF patients did not significantly increase the expression of IL-8 in hUC-MSCs, while the concentration of IL-1β in the serum of ACLF patients was sufficient to significantly increase the expression of IL-8 in hUC-MSCs, suggesting that IL-1β in the serum of ACLF patients may be the key factor inducing the increased expression of IL-8 in hUC-MSCs. The mRNA expression of IKKα, P65, and NF-κB2, the key regulatory factors in the NF-κB signaling pathway, was significantly increased after the stimulation of hUC-MSCs with IL-1β (Fig. 4C). The western blot analysis showed that IL-1β could activate the NF-κB signaling pathway in hUC-MSCs. After IL-1β was neutralized in the serum of dead ACLF patients with a monoclonal antibody against IL-1β, the phosphorylation of P65, a key factor in the NF-κB signaling pathway, was significantly reduced, and the expression of IL-8 in hUC-MSCs was decreased (Fig. 4D). These results suggest that IL-1β in ACLF serum can induce an increase of IL-8 in hUC-MSCs by activating the NF-κB signaling pathway.

### Neutralizing IL-1β in the serum of dead ACLF patients improved the therapeutic effect of hUC-MSCs on ALF

**Fig. 5:**
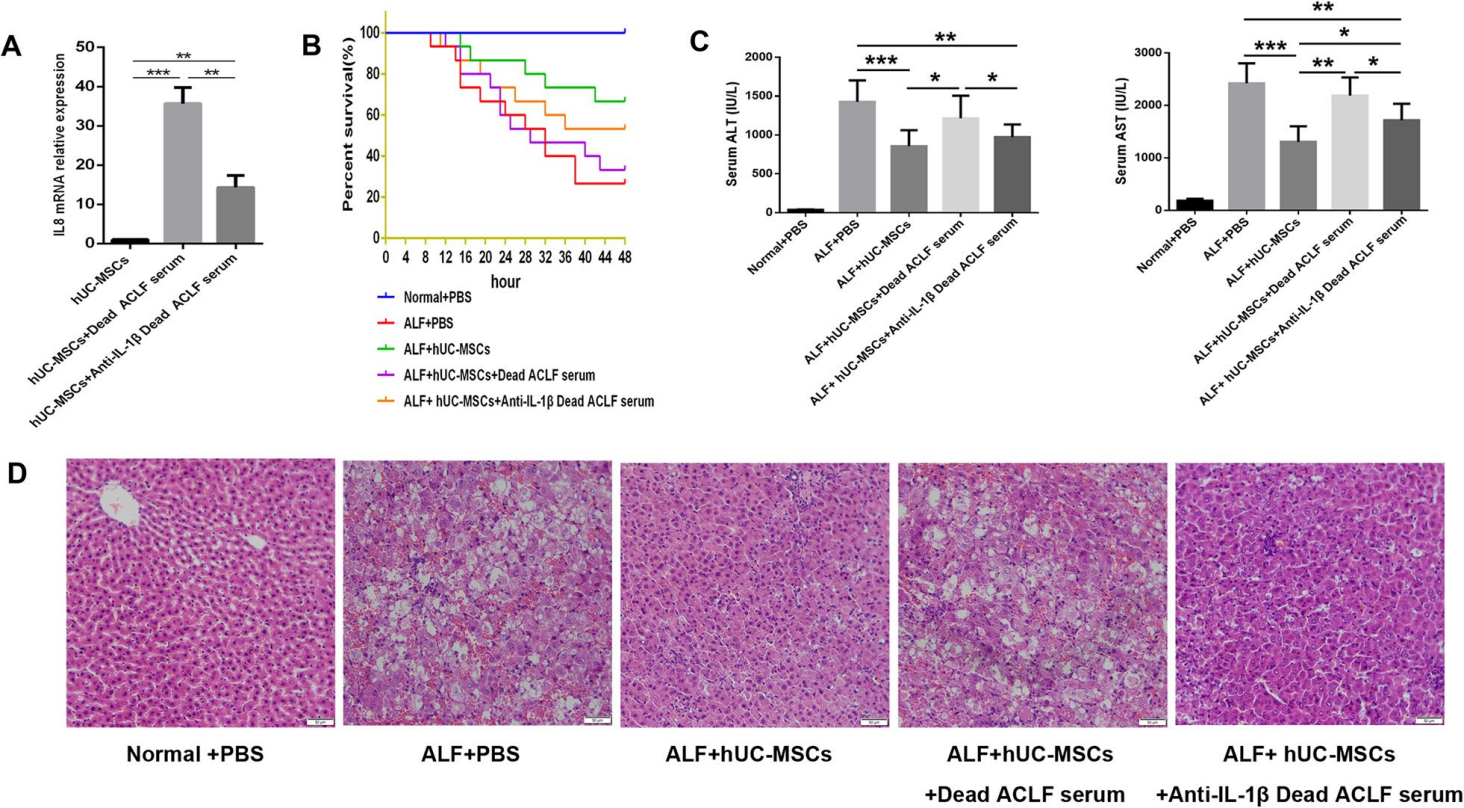
Effect of IL-1β on hUC-MSCs therapy for ALF. **A**The inhibitory effect of IL-1β neutralizing antibody on IL-8 expression in hUC-MSCs. **B** Survival curves of different hUC-MSCs treatment groups (*n* = 15). **C** Serum ALT and AST levels of rats in different treatment groups (*n* = 4 ~ 10). **D** HE staining was used to observe the pathological changes in liver tissue. Scale bar = 50 μm. *P < 0.05; **P < 0.01; ***P < 0.001 (DPI:300)

The high level of IL-1β in the serum of dead ACLF patients can promote the expression of IL-8 in hUC-MSCs, thereby reducing the therapeutic effect of hUC-MSCs in liver failure. Therefore, neutralizing IL-1β in the serum of dead ACLF patients may improve the efficacy of hUC-MSCs on liver failure. The addition of IL-1β neutralizing antibody to dead ACLF serum can significantly reduce the production of IL-8 in hUC-MSCs(Fig. 5A). In the rat ALF model, after the injection of PBS, hUC-MSCs, hUC-MSCs cultured in the serum from dead ACLF patients and hUC-MSCs cultured in the serum from dead ACLF patients with IL-1β neutralization, the survival rate (surviving/total) of ALF rats was 4/15(26.7%), 10/15(66.7%), 5/15(33.3%) and 8/15(53.3%), respectively (Fig. 5B). Liver function ALT/AST (IU/L) was 1425 ± 105.1/2421 ± 144.8, 856.4 ± 72.27/1305 ± 104.6, 1214 ± 97.23/2186 ± 123.3, and 972.2 ± 54.55/1717 ± 104.9, respectively (Fig. 5C). The pathological changes of liver tissue in each group are shown in the figure (Fig. 5D). These results suggest that neutralizing the IL-1β in serum from dead ACLF patients can improve the therapeutic effect of hUC-MSCs on ALF.

## Discussion

At present, the main treatment methods for liver failure include internal medicine treatment, artificial liver and liver transplantation, among which liver transplantation is the most effective treatment [18]. However, due to the lack of donor liver, the high cost of transplantation and the serious immune rejection after transplantation, its wide application is restricted [19]. Therefore, it is still an urgent clinical problem to find a new method for the effective treatment of liver failure. In recent years, MSCs have been used in the treatment of a variety of diseases, including liver failure, cirrhosis and fatty liver [20–23]. Most studies focus on the mechanism of how MSCs can effectively treat liver failure, but there is a lack of research on the mechanism of their poor efficacy in the treatment of liver failure. The immunomodulatory function of MSCs is one of the most important reasons for their role in the treatment of various diseases [24], and this immunomodulatory function will be affected by various inflammation-related factors in patients’ serum [25]. The cytokines in the serum of patients with liver failure are complex, and the levels of inflammatory factors are generally elevated. It is unclear how the inflammatory environment of liver failure affects MSCs function. Therefore, this study aims to explore the possible reasons why the serum of ACLF patients affects the efficacy of MSCs and to provide evidence for how to choose ACLF patients who are suitable for stem cell therapy.

Compared with the normal culture state, the proliferation of hUC-MSCs was inhibited under ACLF serum treatment, and the inhibitory effect of serum from surviving ACLF patients was more obvious. This proliferation-inhibitory effect may be a stress response to hUC-MSCs entry into ACLF serum. However, the growth of hUC-MSCs stimulated by the serum of dead ACLF patients may be related to high levels of some inflammatory factors. Studies have shown that both TNF-α and IL-1β can promote the proliferation of stem cells in vitro [26, 27]. Similarly, serum in the disease state promotes stem cell proliferation more than healthy human serum [28, 29].

The expression profile of hUC-MSCs was changed after cultured with ACLF serum. The mRNA expression levels of 22 factors in hUC-MSCs cultured in the serum of dead ACLF patients were compared with those in hUC-MSCs cultured in the serum of surviving ACLF patients. The mRNA expression levels of IL-10, IL-6, IL-8, IFN-γ, HGF, CXCR4 and MCH-II showed statistically significant differences between the two groups, and the expression level of IL-8 showed the most significant change. The expression level of IL-8 in hUC-MSCs cultured in the serum of dead ACLF patients was significantly higher than that of hUC-MSCs cultured in the serum of surviving ACLF patients. The level of IL-8 in hUC-MSCs treated with CHB and ACLF serum was further detected by ELISA, and it was found that the expression of IL-8 in hUC-MSCs increased with the aggravation of the disease. At the same time, we detected the expression level of IL-8 in the serum of healthy people, CHB patients and ACLF patients by ELISA and found that the level of IL-8 in serum increased with the aggravation of the disease, which was consistent with the results reported in the literature; that is, the level of IL-8 in the serum of ACLF patients was significantly higher than that of healthy people, and the level of IL-8 in serum was related to liver injury and the severity of the disease [30, 31].

IL-8 belongs to the C-X-C subfamily of chemotactic cytokines and is a chemotactic factor of various cell origins. IL-8 activates neutrophils, chemotaxis to T lymphocytes and stimulates basophil granulocytes, and is involved in the occurrence and development of a variety of diseases [32]. IL-8 can aggravate the lesions of patients with psoriasis and is the main chemokine of neutrophil aggregation in lesions [33]. The level of IL-8 in the synovial fluid of patients with rheumatoid arthritis is significantly increased, and the level of IL-8 is positively correlated with the severity of rheumatoid arthritis [34]. ACLF serum can significantly increase the expression level of IL-8 in hUC-MSCs, but the effect of IL-8 on the improvement of hUC-MSCs in liver failure remains unclear. We reduced the expression of IL-8 in hUC-MSCs by siRNA interference and found that interfering with the expression of IL-8 in hUC-MSCs cultured in the serum of dead ACLF patients could improve the therapeutic effect of hUC-MSCs on ALF in rats. This result suggested that the high expression of IL-8 could reduce the therapeutic effect of hUC-MSCs on ALF in rats. High levels of IL-8 in serum suggest poor prognosis in liver failure [35, 36]. The most classical role of IL-8 is to chemotactic and activate neutrophils in various diseases [37]. Previous studies have shown that high concentrations of IL-8 in serum lead to increased infiltration of neutrophils in liver tissue, thereby exacerbating liver tissue damage [38]. IL-8 has been detected in microvesicles derived from stem cells and tumor cells [39, 40]. Therefore, IL-8 can be secreted from cells through microvesicles. However, it remains unclear whether stem cells secrete IL-8 exclusively through microvesicles.

TNF-α and IL-1β have been reported to increase the expression of IL-8 in endothelial cells and liver cells [41]. In this study, it was found that both TNF-α and IL-1β could activate the expression of IL-8 in hUC-MSCs, and the activation effect of IL-1β was much stronger than that of TNF-α. The levels of TNF-α and IL-1β in the serum of healthy people, CHB patients and ACLF patients were further determined. The levels of TNF-α and IL-1β in serum increased with the aggravation of the disease. Although the expression level of TNF-α in ACLF serum was significantly higher than that of healthy peoples and CHB patients, the concentration level of TNF-α in ACLF serum was not sufficient to cause the increase of IL-8 expression in hUC-MSCs in vitro cell experiments. Therefore, the significant increase in the expression level of IL-8 in hUC-MSCs caused by serum from ACLF patients may be mainly due to the IL-1β in serum.

IL-1β is a major regulator of the inflammatory response, and it can promote lymphocyte proliferation and activate monocytes and natural killer cells [42]. Meanwhile, IL-1β also induces the production of other inflammatory cytokines (such as IL-6 and TNF-α), chemokines, adhesion factors and acute phase proteins [43]. The biological effect of IL-1β is mainly achieved through its postreceptor signal transduction, and the signal transduction pathways of IL-1β include MAPK, NF-κB, AP-1, NF-IL6, JNK and P38. NF-κB, C/EBP and AP-1 binding sites near IL-8 genes are major cis-acting elements regulating IL-8 promoter function [44]. Previous studies have shown that activation of the NF-κB signaling pathway can lead to increased expression of IL-8 in cells [45]. NF-κB is an evolutionarily highly conserved transcription factor that plays a key role in many biological processes. NF-κB is usually present in the cytoplasm in the form of a dimer, and it is activated by stimulating factors (IL-1β, TNF-α and LPS) and then enters the nucleus to regulate the transcriptional expression of a variety of genes, thus causing a large number of inflammatory cells to infiltrate the inflammatory site, leading to the continuation or amplification of inflammation. In this study, after IL-1β treatment of hUC-MSCs, Q-PCR was used to detect the changes in the expression of factors related to the IL-1β signal transduction pathway in hUC-MSCs, and it was found that the P65 expression in the NF-κB pathway was the most significantly different. Western blotting was used to further verify the relationship between IL-1β, NF-κB and IL-8, and it was found that IL-1β could regulate the expression of IL-8 in hUC-MSCs through the NF-κB signaling pathway. Many studies have shown that INF-γ combined with TNF-α or IL-1β can enhance the immunosuppressive effect of stem cells, but TNF-α or IL-1β alone cannot exert a similar effect [46, 47]. The immunomodulatory capacity of stem cells is plastic, and they can exhibit either anti-inflammatory or proinflammatory phenotypes [48]. In this study, we found that IL-1β in the serum of dead ACLF patients may induce hUC-MSCs to develop into a proinflammatory phenotype, and that neutralizing IL-1β can improve the efficacy of hUC-MSCs.

## Conclusions

**Fig. 6:**
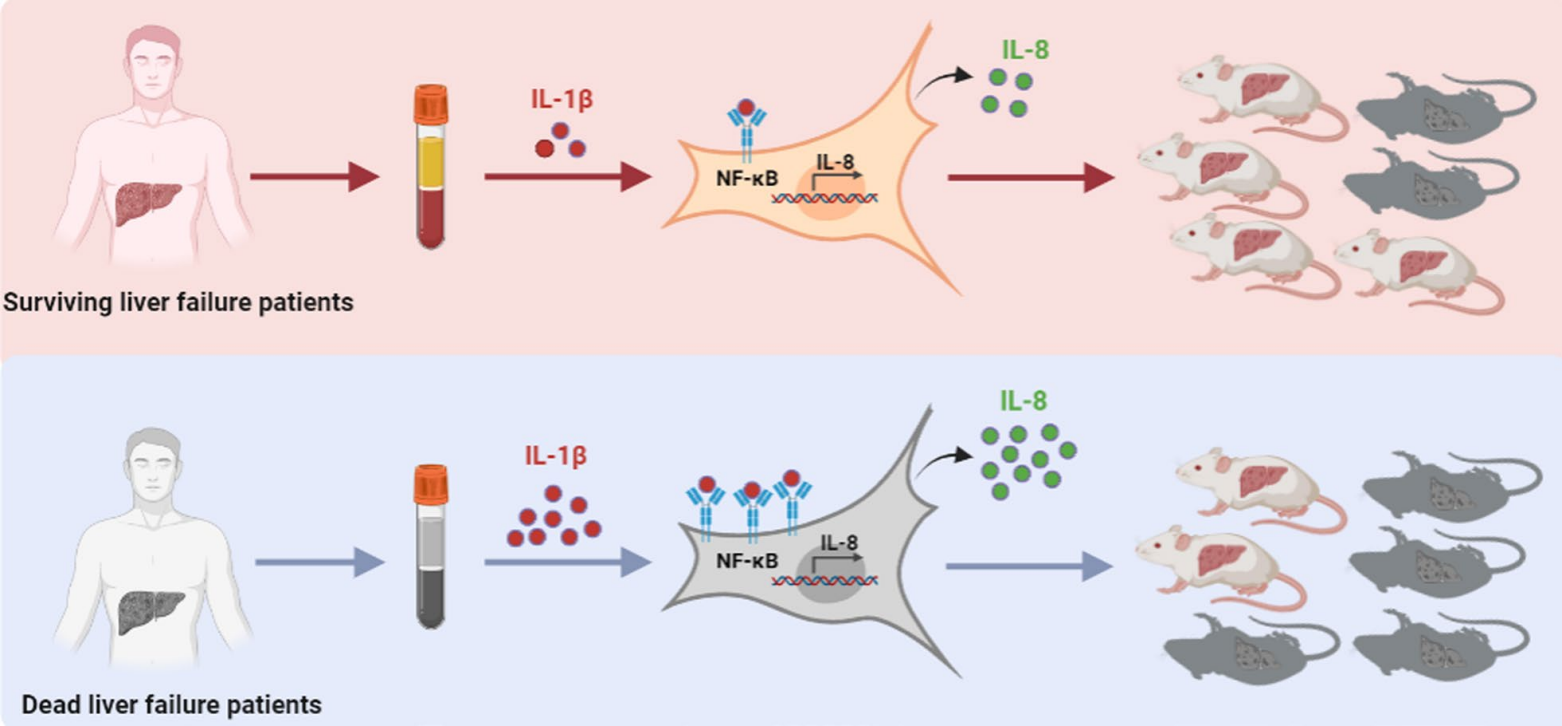
The potential mechanism by which ACLF patient serum affects the therapeutic effect of hUC-MSCs on liver failure. IL-1β was significantly higher in the serum of dead ACLF patients than in the serum of surviving ACLF patients. The high level of the inflammatory factor IL-1β in serum from dead ACLF patients can promote the expression of IL-8 in hUC-MSCs through the NF-κB signaling pathway, thereby reducing the effect of hUC-MSCs in the treatment of liver failure.The figure was drawn by the author of this study (DPI:300)

Serum from ACLF patients can affect the cytokine secretion of hUC-MSCs. The high level of the inflammatory factor IL-1β in serum from ACLF patients can promote the expression of IL-8 in hUC-MSCs through the NF-κB signaling pathway, thereby reducing the effect of hUC-MSCs in the treatment of liver failure (Fig. 6). Interfering with the expression of IL-8 in hUC-MSCs or neutralizing IL-1β in serum from ACLF patients can improve the efficacy of hUC-MSCs. Therefore, the level of inflammatory factors (such as a high level of IL-1β) in liver failure patients can affect the efficacy of stem cells, but improving the inflammatory environment in liver failure patients prior to the application of stem cell therapy may improve the efficacy of stem cells.

### Supplementary Information

**Additional file 1**. **Fig s1**: Identification of hUC-MSCs. (**A**) Observation of cell morphology under an optical microscope. (**B**) Detection of adipogenesis and osteogenesis capacity of hUC-MSCs. (**C**) Glycogen staining and indocyanine green uptake were used to determine the function of hepatocyte-like cells. (**D**) The expression levels of AFP, CK18 and ALB in hUC-MSCs were detected by Q-PCR at different induction times. (**E**) Analysis of hUC-MSCs surface markers by flow cytometry. **Fig s2**: Original picture of the full-length blots. (**A**) p-NF-κB-p65. (**B**) NF-κBp65. (**C**) IL-8. (**D**) GAPDH. **Table s1**: Primer sequences of 22 factors in hUC-MSCs. **Table s2**: Primers sequence of key factors in IL-1β related signaling pathway. **Table s3**: Interference sequence of siRNA -IL-8.

## Data Availability

The datasets used during the current study are available from the corresponding authors on reasonable request.

## Abbreviations

hUC-MSCs: Human umbilical cord MSCs
IL-1β: Interleukin-1β
IL-8: Interleukin-8
ACLF: Acute-on-chronic liver failure
ALF: Acute liver failure
MSCs: Mesenchymal stem cells
IFN-γ: Interferon γ
TNF-α: Tumor necrosis factor α
IDO: Indoleamine 2,3-dioxygenase 1
IL-10: Interleukin-10
TGF-β: Transforming growth factor β
CHB: Chronic hepatitis B
ALT: Alanine aminotransferase
AST: Aspartate aminotransferase
d-GalN/LPS: d-galactosamine/lipopolysaccharide
NF-κB: Nuclear factor kappa-B
PBS: Phosphate buffer solution
