# Supplementary material for: The high level of IL-1β in the serum of ACLF patients induces increased IL-8 expression in hUC-MSCs and reduces the efficacy of hUC-MSCs in liver failure
Source: Stem Cell Res Ther. 2023 Aug 30;14:231. doi: 10.1186/s13287-023-03455-9 (PMC10468895; doi:10.1186/s13287-023-03455-9)
Supplement: Supplementary file 1 — Additional file 1. Fig s1: Identification of hUC-MSCs. (A) Observation of cell morphology under an optical microscope. (B) Detection of adipogenesis and osteogenesis capacity of hUC-MSCs. (C) Glycogen staining and indocyanine green uptake were used to determine the function of hepatocyte-like cells. (D) The expression levels of AFP, CK18 and ALB in hUC-MSCs were detected by Q-PCR at different induction times. (E) Analysis of hUC-MSCs surface markers by flow cytometry. Fig s2: Original picture of the full-length blots. (A) p-NF-κB-p65. (B) NF-κBp65. (C) IL-8. (D) GAPDH. Table s1: Primer sequences of 22 factors in hUC-MSCs. Table s2: Primers sequence of key factors in IL-1β related signaling pathway. Table s3: Interference sequence of siRNA -IL-8. [file 13287_2023_3455_MOESM1_ESM.pdf]

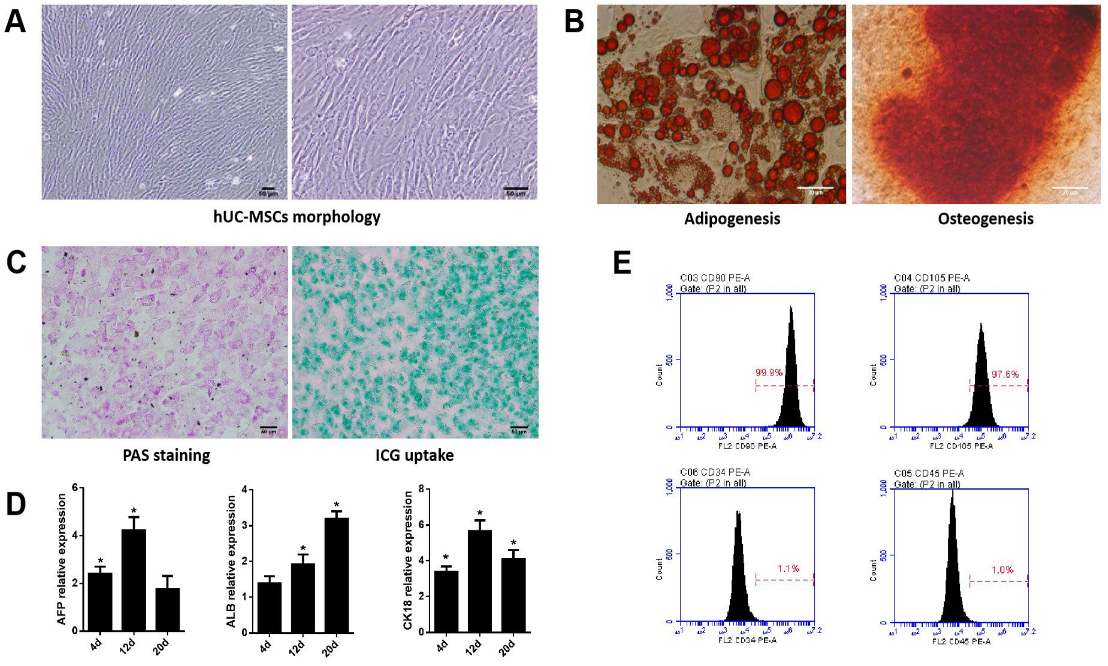

Fig s1 Identification of hUC-MSCs. (A) Observation of cell morphology under an optical microscope. (B) Detection of adipogenesis and osteogenesis capacity of hUC-MSCs. (C) Glycogen staining and indocyanine green uptake were used to determine the function of hepatocyte-like cells. (D)The expression levels of AFP, CK18 and ALB in hUC-MSCs were detected by Q-PCR at different induction times.(E) Analysis of hUC-MSCs surface markers by flow cytometry.

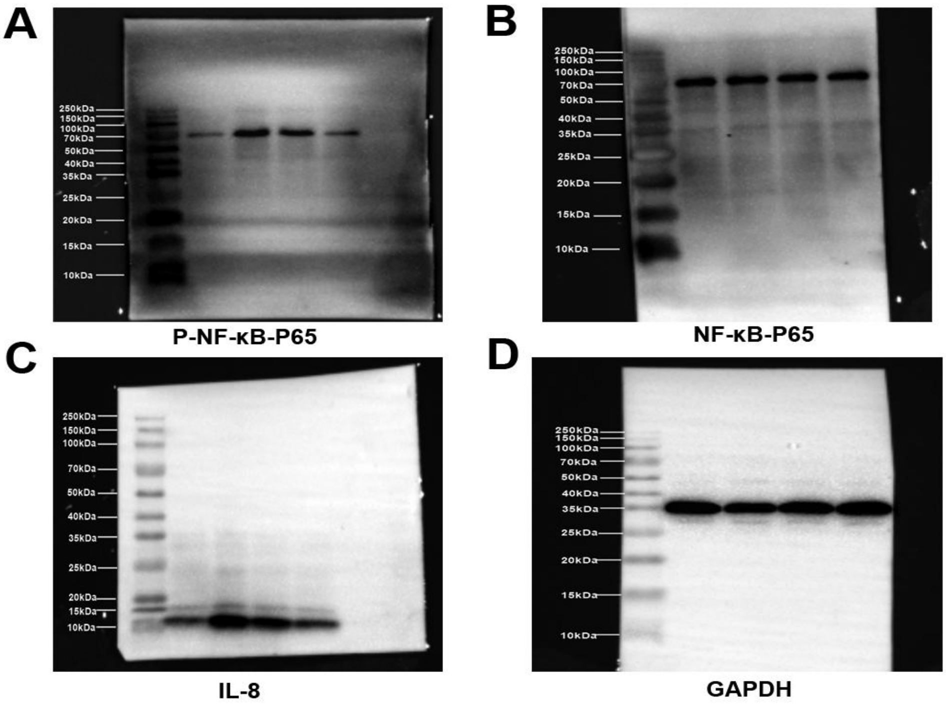

Fig s2 Original picture of the full-length blots. (A) p-NF-κB-p65. (B) NF-κB-p65. (C) IL-8. (D) GAPDH.

Table s1 Primer sequences of 22 factors in hUC-MSCs

Primer name	5′ -primer sequence-3′	Primer name	5′ -primer sequence-3′
GAPDH-F	GGAGCGAGATCCCTCCAAAAT	MIP-1a-F	TGCCAAACAGCCACACT
GAPDH-R	GGCTGTTGTCATACTTCTCATGG	MIP-1a-R	GGGGACAGGGGAACTCT
IDO1-F	TGCCAACTCTCCAAGAAAC	MCP-1-F	TTTTCCCCTAGCTTTCCC
IDO1-R	GCAGTCTCCATCACGAAAT	MCP-1-R	GCAATTTCCCCAAGTCTCT
SDF1a-F	CCAACGTCAAGCATCTCA	CXCR4-F	CAAGCAAGGGTGTGAGTTT
SDF1a-R	CACTTTAGCTTCGGGTCAA	CXCR4-R	AGCATAGAGGATGGGGTTC
MCH-II-F	CCAGTTTTACGGTCCCTCT	IL2-F	AACTCACCAGGATGCTCACA
MCH-II-R	GCCACAGCCATGTTTCTC	IL2-R	GCACTTCCTCCAGAGGTTTGA
IL4-F	GCATCGTTAGCTTCTCCTG	M-CSF-F	GTGGGAGAGAGCAAGGGA
IL4-R	ATATCGCACTTGTGTCCGT	M-CSF-R	GGGTTTGGGAGTGCAGAC
VEGF-F	CTGGGCTGTTCTCGCTT	IL-1β-F	CACGATGCACCTGTACGA
VEGF-R	CCCCTCTCCTCTTCCTTCT	IL-1β- R	AACACCACTTGTTGCTCCA
IL8-F	GACATACTCCAAACCTTTCCA	IL10-F	GACTTTAAGGGTTACCTGGGTTG
IL8-R	AACTTCTCCACAACCCTCTG	IL10-R	TCACATGCGCCTTGATGTCTG
IFN-γ-F	ACTTGAATGTCCAACGCAA	IL6-F	CCTGAACCTTCCAAAGATGGC
IFN-γ-R	CGAAACAGCATCTGACTCC	IL6-R	TTCACCAGGCAAGTCTCCTCA
ICAM1-F	ACCTGCCTTTCCCCAGA	HGF-F	GCTATCGGGGTAAAGACCTACA
ICAM1-R	ACCGCTGAGTGTCATTGTG	HGF-R	CGTAGCGTACCTCTGGATTGC
FGF2-F	GAGGACCCATAAGAGTTCACA	TNFa-F	GAGGCCAAGCCCTGGTATG
FGF2-R	TGCAGCCTTACCCAATCTA	TNFa-R	CGGGCCGATTGATCTCAGC
SCF -F	GCTCACCCATATCCCACTAT	TGFβ-F	CTAATGGTGGAAACCCACAACG
SCF -R	GTTCTTAACTTCTTCCTGCGA	TGFβ-R	TATCGCCAGGAATTGTTGCTG
LIF-F	CCAGCATTGTGTTGTGAGG		
LIF-R	TGTTCCAGGGCGCTATTT		

Table s2 Primers sequence of key factors in IL-1β related signaling pathway

Primer name	5′ -primer sequence-3′
NF-KB-P65_F	ATGCGCTTCCGCTACAA
NF-KB-P65_R	GTGACCAGGGAGATGCG
AP1_F	AGCGGACCTTATGGCTACA
AP1_R	GTGATGTGCCCGTTGCT
MAPK_F	TACGACCCGAGTGACGA
MAPK_R	TCTGAGCCCTTGTCCTGA
JUK_F	TCTCCAACACCCGTACATC
JUK_R	CCTCCAAGTCCATAACTTCCT
P38_F	TCCAGACCATTTCAGTCCA
P38_R	CGTCCAACAGACCAATCAC
IKKa_F	CACAGACCTTCAGATCACTCC
IKKa _R	TGCTTACAGCCCAACAACT
NF-KB-P52_F	GCACCTAGCCATCATCCAC
NF-KB-P52_R	TACCCGCAGCAGAAAGC

Table s3 Interference sequence of siRNA-IL-8

siRNA name	5′ -interference sequence-3′
siRNA-IL-8 Sense	GCCAGAUGCAAUACAAGAUTT
siRNA-IL-8 Antisense	AUCUUGUAUUGCAUCUGGCTT
